# Standardized Ixodid Tick Survey in Mainland Florida

**DOI:** 10.3390/insects10080235

**Published:** 2019-08-01

**Authors:** Gregory E. Glass, Claudia Ganser, Samantha M. Wisely, William H. Kessler

**Affiliations:** 1 Department of Geography and Emerging Pathogens Institute, University of Florida, Gainesville, FL 32610, USA; 2 Department of Wildlife and Ecology and Conservation, University of Florida, Gainesville, FL 32610, USA

**Keywords:** ixodid ticks, *Ixodes*, Amblyomma, Dermacentor, field survey, geographic distribution, range, bias

## Abstract

A statewide survey of questing ixodid ticks in mainland Florida was developed consistent with U.S. CDC standards to maximize the amount of epidemiologic and environmental data gathered. Survey sites were stratified by climatic zones and proportional to recognized land cover categories. A total of 560 transects on 41 sites within the state were sampled repeatedly by flagging between 2015 and 2018. Four tick species were collected; *Amblyomma americanum, Amblyomma maculatum, Ixodes scapularis* and *Dermacentor variabilis*. All species were more commonly found in northern and central regions of the state than in southern and western regions. Adult *I. scapularis* were active from autumn through spring and complementary to adult *A. americanum* and *D. variabilis*. Standardized survey methods help reduce sampling biases and better characterize risk from the species surveyed. However, differences in the attractiveness of collection methods for different tick species makes cross-species comparisons a continuing challenge.

## 1. Introduction

The impacts of ticks and tick-borne pathogens (TBP) have come into increasing focus during the past several decades [1,2,3]. Because many of the zoonotic TBPs are reported predominantly in one or a few species of ticks, the distributions and abundances of the various vectors have major roles in characterizing the spatial extent of human and animal risk [3]. In this regard, regional or national estimates of vector distributions through species distribution models (SDMs) have provided a critical tool to broadly characterize recent changes in patterns of risk [4,5]. The U.S. Centers for Disease Control and Prevention has proposed standardized survey protocols to gather data on potential pathogen vectors with the goal of educating public health entomologists and biologists on what types of information can be extracted from various survey methods and how the surveys should be performed [6].

In North America, the state of Florida, located in the southeastern-most portion of the continental United States, represents a unique situation for tick vectors and TBPs. It is the 22nd largest state geographically—covering approximately 170,300 sq km, but the third largest in terms of the human population [7] with an estimated 21 million people in 2017 and an annual growth rate of 1.5% [7]. Additionally, significant portions of the residential population engage in outdoor occupations and the generally mild climate makes outdoor recreation common [8]. For example, in 2017, agriculture, including ranching and timber harvesting, engaged more than 2 million residents and represented one of the top two economic resources for the state [8]. More than 116.5 million people also visited for recreational activities [8]. Additionally, the state’s six climate zones range from subtropical, in the north, to tropical, in the south [9,10]. These conditions are associated with a single rainy season between June and September when an average of 705 mm of rain falls [11]. As such, the combination of social, climatic and environmental conditions raises the potential for tick exposures and transmission of their pathogens.

Up to 20 species of hard-bodied ticks have been reported in the state [12]. Records from the state extend to the early 1900s [13]. Much of the more recent information has been built from collaborations among agencies working with convenience samples from the public so that detailed characterizations of the geographic distributions of these arthropods within the state are subject to vagaries of survey methods, geographic extents of surveys, and access to materials. National and regional analyses that relied on information from various collection sources [4,5] indicate that selected species should be widely distributed in the region. Recently, Kessler and colleagues [14] developed a more spatially detailed analysis of *Amblyomma americanum* within the state and reported that the predicted species distribution was anticipated to be substantially more nuanced, with much of the spatial heterogeneity in the species distribution associated with landcover, temperature, and precipitation patterns, which was obscured in national-level analyses which focused on regional climatic variability.

At the appropriate scale, national-level data provide critical information on the broad geography of various species in a cost-effective manner, help raise hypotheses concerning drivers of TBP risk [4,5] and inform national level policies. However, at the local level, where detailed investigations or interventions might be planned, county-level predictions may be too broad. Additionally, passive surveillance records or historical documentation may be spatially/environmentally biased and cause SDMs to unalterably misrepresent the real distributions [15].

To develop an updated spatial database of human-seeking tick species in the state, a standardized survey protocol was developed and implemented by applying CDC protocol recommendations [6]. The survey focused on mainland Florida but did not sample the Florida Keys, which is a seventh, separate climatic region. The goal was to use a collection scheme that targeted major biotopes (= habitat types) throughout the mainland so that these data could be extrapolated more broadly using SDMs, incorporating more extensively monitored environmental covariates [16]. Surveys were conducted from October 2015 through December 2018. The goal was to establish a format to deal with external validation issues of SDM’s for tick vectors in Florida. Specifically, it attempts to develop an approach for datasets that can act to predict vector distributions.

### 1.1. Historical Surveys in Florida

Prior to initiating the survey, a literature and museum review of ticks reported in the state was performed. These results are briefly outlined here. Some of the earliest reports of ixodid tick collections in Florida were from Nathan Banks [17,18], beginning in 1901, who relied on several collectors in regions of southern and north central parts of the state, including Lake Worth, Palm Beach, Punta Gorda and Enterprise [17]. Shortly after, Hooker et al [19] extended information on the bionomics of the major North American tick species, including those of Florida. Subsequent surveys in the 1930s and late 1940s, provided more extensive information on the geographic distributions. Additional, later reports by Bishopp and Trembley [20] expanded the characterization of the species recognized at that time. Many of the specimens that formed the bases for these reports were retained in the U.S. National Tick Collection (Statesboro, GA USA) [12]. The spatial distribution of 13 ixodid tick species in Florida was summarized in a 1951 unpublished Master’s thesis by D.J. Taylor [21], that provided information on county-level species’ distributions and added species records in 49 counties, while A.J. Rogers [22] detailed the bionomics and activity patterns of ixodid ticks within the northern region of the state.

Localized reports within the state continued through the 1960s into the 1980s. Reports of geographically extensive surveys became more common after that time. Greiner and colleagues [23] marked the beginning of more recent, intensive and widespread surveys in the state, with a study of ticks on nearly 650 boars in the southern portion of the state. Findings indicated four predominant species, *Dermacentor variabilis*, *Amblyomma maculatum*, *Ixodes scapularis* and *Amblyomma americanum.* The latter two species were represented by less than 10 individuals apiece. Cilek and Olson [24] flagged for questing ticks in the panhandle (Northwestern Florida) of the state. They surveyed several habitats during weekly surveys for two years. They reported the same four species as Greiner and colleagues [23] but in reverse order of abundance. Burroughs and colleagues [25] used domestic animals from 22/67 counties in the state, adding *Rhipicephalus sanguineus* to the previously sampled species. It was the only species that they found throughout the state and was the most abundantly collected tick reported. All but one of the *R. sanguineous* were collected from domestic dogs (one was from a domestic cat). The remaining four species were restricted to pets from northern and central portions of the state. The following year, Hertz and colleagues [26] reported on a combination of collection kits from participating wildlife hunters, and materials archived by the Florida Fish and Wildlife Conservation Commission. This survey yielded animals from two-thirds of the state’s counties and added recent records for *Ixodes affinis* and *Ixodes texanus*, although they were less than 1% of ticks recovered. They reported the same rank order of abundance as Cilek and Olson [24]. Recently, Merrill and colleagues [27,28] performed a limited survey on small and moderate-sized mammals in the south-central region, confirming Greiner [23] results, and then conducted a more localized study. The local study was notable for comparing the two common survey methods for ticks; flagging and examination of wildlife. The study compared ticks collected on 84 km of transects with examination of 316 boar. The study collected almost exclusively adult *Amblyomma maculatum*, but also included adult *A. auricularium, D. variabilis* and *I. scapularis* from boar—one nymph was collected. Flagging yielded nearly 3000 immatures but sampled fewer adults and did not sample any *D. variabilis*.

The studies that were geographically wide ranging noted that questing ticks were more abundant in the north and central region of the state and decreased towards the southern areas [21,22,25,26]. However, several species were focally collected in southern regions adjacent or within portions of the Everglades [29]. Some of this variation may reflect differences in survey methods but was also presumed to reflect aspects of local variation in habitat and host abundance [29].

### 1.2. Health and Disease

Current discussions about tick presence and abundance often reflect concerns about pathogen transmission to humans, domestic animals and livestock [30]. Prior to the 1970s, the primary human pathogen associated with TBD in the state was *Rickettsia rickettsiae* and Rocky Mountain Spotted Fever, which was occasionally reported [21]. Taylor described two human cases from Northern Florida in 1947 [21]. Tularemia was recognized as associated with *Haemaphysalis leporis-palustris* and bovine babesiosis in cattle and was also linked with *Boophilus annulatus.* Otherwise, ixodid ticks were usually characterized as pests that might physically damage domestic animals or people, rather than being significant health threats associated with microorganisms [20,21].

However, the association of diseases with *Babesia microti* and *Borrelia burgdorferi* nearly 50 years ago [31,32,33] focused attention on the roles of ticks as vectors of bacterial and viral agents. Subsequently, there was increasing interest and a steady characterization of bacterial and viral pathogens associated with tick vectors. We presumed potential pathogen risks from collected tick species during this study could follow previous, general descriptions [6]. However, recent developments of modern molecular diagnostics have opened opportunities to further rapidly identify and characterize a diverse array of agents associated with various tick species e.g., [34].

## 2. Materials and Methods

To characterize the distributions of selected ixodid tick species in Florida, sampling was performed over the geographic extent of the mainland. The diverse land cover and variation in climate of this region encouraged a sampling scheme to address the range of possible environments that might impact the occurrence/abundance of tick species. A standardized collection and data survey strategy was developed. Two major factors, based on the literature, were used to stratify collections; climate and land cover [35]. Categories of land cover across the six major climate zones of mainland Florida [10,36] were targeted with the goal of sampling environmental conditions proportional to the recognized cover classes within the state. The Florida Cooperative Land Cover (FCLC) Database v2.3, produced in late 2012, was selected for the sampling frame [37]. The database “…uses the Florida Land Cover Classification System (FLCS), a hierarchical classification system developed by the Florida Fish & Wildlife Conservation Commission.” [37]

Within the 37 second-order major land cover categories identified within the state that had more than 1% areal coverage, sites controlled by federal, state, local agencies and private owners were identified, and permissions obtained to survey for questing ticks throughout the year. Within each site, distinct biotopes were identified during field surveys. Subsequently, at least two transects separated by 200 m or more (at their midpoints) were identified in the major local biotopes and surveyed for ticks. The numbers of sites in each climatic zone were targeted to be proportional to the geographic extents of the six recognized climatic zones.

Surveys were conducted by flagging with a light-colored flannel cloth attached to a pole by spring-loaded clips. Collectors swept the leaf litter, standing vegetation and fallen natural structures while walking transects. Approximately every 10 m, the cloths and collectors were examined for attached life stages of ticks, which were removed and stored in 100% ethanol for identification in the laboratory. Ticks were identified using morphological keys and a microscope [14,16]. The target length for transects was 150 m, as a compromise between distance sampled and time available. At the end of the transect, the cloths were removed from the poles and stored in labeled sealed bags for later examination to ensure complete collection and to prevent carryover contamination to other locations. Surveys were repeated four to six times during the year, depending on the availability of access and weather conditions at individual locations.

Each time a survey was conducted on a transect, the data were recorded with a personal digital assistant using a customized digital data form created in DoForms [38]. Recorded data included county and nearest town/city, date and the GPS coordinates of the start, mid-point and end of the transects, as well as digital images of the GPS locations which were collected to validate the local FCLC classification and confirm transects were completed. Data were subsequently uploaded and stored at the UF Emerging Pathogens Institute (UF EPI) computing environment. These data were then associated within a Geographic Information Systems environment (ArcGIS version 10.6.1) at UF EPI and combined with other environmental data layers for further analyses.

Basic summary statistics were calculated using Statistix version 10.0. Time (measured by the number of times individual sites were surveyed) until the first of any tick species was found was plotted as a histogram to identify the detection sensitivity using flagging. The goal was to identify the sampling effort required to rule out locations and conditions that were unlikely to yield questing ixodid ticks. The number of transects in each land cover class was compared with the geographic extents of the land classes. Categories with less than 1% coverages were grouped as ‘other’ and a 2 × n Chi Square test was performed to determine if there were significant differences in the number of transects in land use classes and their classes’ areal coverage.

Seasonality of adults of the three most common tick species (*I. scapularis, A. americanum* and *D variabilis*) was characterized by the proportion of transects yielding one or more individuals of each species during each month of the year. The exception was February when surveys were not conducted.

Ordinary kriging was used to estimate the distance to independence among samples sizes of collected adult ticks on the transects to identify the extent of spatial clustering of observed tick abundances. The semivariogram was calculated using the geostatistical wizard from the geostatistical analyst toolbox in ArcGIS 10.6.1, as implemented for ordinary kriging. The semivariogram, defined as: $\gamma \left(x_{i},x_{j}\right)=\frac{1}{2}var\left(Z\left(x_{i}\right)-Z\left(x_{j}\right)\right),$ where *var* is the variance between values at two locations *X_i_* and *X_j_*, and an empirical semivariogram was fit to estimate the nugget (n), sill (s) and range (r) parameters. Several model functions were tried, before a Gaussian variogram model was chosen. The Gaussian form of the empirical variogram model is: $\gamma \left(h\right)=\left(s-n\right)\left(1-\exp\left(-\frac{h^{2}}{r^{2}a}\right)\right)+n1_{\left(0,\infty \right)}\left(h\right)$, where h is the lag distance.

To compare alternative sampling strategies, two shorter term surveys were performed. We compared dry ice traps with flagging. Adjacent to selected flagging sites, dry ice was placed on an unused flag laid flat on the ground [39]. The dry ice trap was left in place while flagging was conducted and was then collected and ticks tallied. Because of the reported predilection of adult *A. americanum* for dry ice traps, these surveys were performed during seasons and at sites where the species had previously been sampled. The second method surveyed wild boar that were collected as part of a herd culling effort in two counties of Southern Florida. Dead wild boar that were brought to the survey center were examined around the head, especially the ears, neck and chin, as well as the upper legs and chest [23,27]. These surveys primarily represented a convenience sample focusing in a geographic region where previous flagging yielded few questing adult ticks. The purpose was to evaluate earlier reports that wildlife provide more sensitive detection than active surveillance by researchers [27]. Thus, even if wildlife do not detect different species, compared to flagging, the abundance of the species may be higher for wildlife [23].

## 3. Results

A total of 560 distinct GPS coordinates were recorded for the centroids of the transects that were located at 41 sites within mainland Florida. These sites ranged from north of Pensacola, in Northwestern Florida to north of Jacksonville, in the northeast, to south of Miami in the southeast to south of Tampa in the southwest (Supplementary Materials Figure S1). The average density of sites per climatic zone was 1 site/3866.1 sq km, ranging from 1 site/2667.7 sq km in zone 3 to 1 site/4949.9 sq km in zone 6. Under-sampling in zone 6, along the Southern Atlantic coast was driven by the urban and suburban development of coastal South Florida, which limited the numbers of available sites.

**Figure 1:**
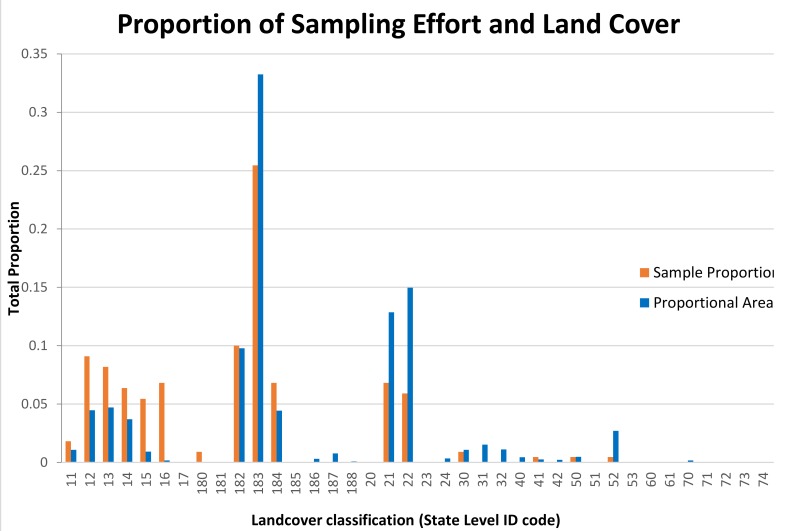
Proportion of transects in various landcover categories (orange) compared with geographic extent (blue) within Florida. There was no significant difference in the landcover classes surveyed compared with their areal extents.

The proportions of transects distributed among the land cover classes of FCLC were not significantly different from the occurrences of the habitat classes within the state (*p* > 0.05) (Figure 1). There was a tendency for rural croplands (class 183), freshwater wetlands (classes 21–22) and the shoreline (class 52) to be underrepresented, with forests or shrublands (classes 11–16) to be overrepresented, but the differences were not statistically significant.

A total of 1956 surveys were conducted on 560 transects at the 41 sites during the study. Based on the GPS start, middle and stop locations, the nominal length of the transects averaged 153 m ± 2 m. This represented 299 km of flagging. The time to conduct a complete survey cycle of the mainland (Supplementary Materials Figure S1) was typically 1–2 weeks, depending on weather conditions.

During these surveys, a total of 793 adult ticks and 1240 nymphal ticks were collected and catalogued. In addition, pools of larval ticks were collected and stored. Four species of ticks were identified; *Dermacentor variabilis, Ixodes scapularis, Amblyomma americanum* and *Amblyomma maculatum*. *I. scapularis* (29.5%) and *A. americanum* (63.2%) predominated in the adult samples (Table 1). Nearly all (>95%) nymphs were identified as *A. americanum*, although a small fraction of *A. maculatum* nymphs also were noted.

The abundance of many ticks often is reported to be spatially overdispersed [40]. This was consistent with our observations. Spatial clustering of ticks collected by flagging occurred at very local levels so that observations quickly became spatially independent. Numbers of *I. scapularis* and *A. americanum* were sufficient to determine the distance to independence among samples. Based on simple kriging, the calculated major range (distance to independence between samples) for *I. scapularis* was 91 meters, while for *A. americanum* it was 256 meters.

**Figure 2:**
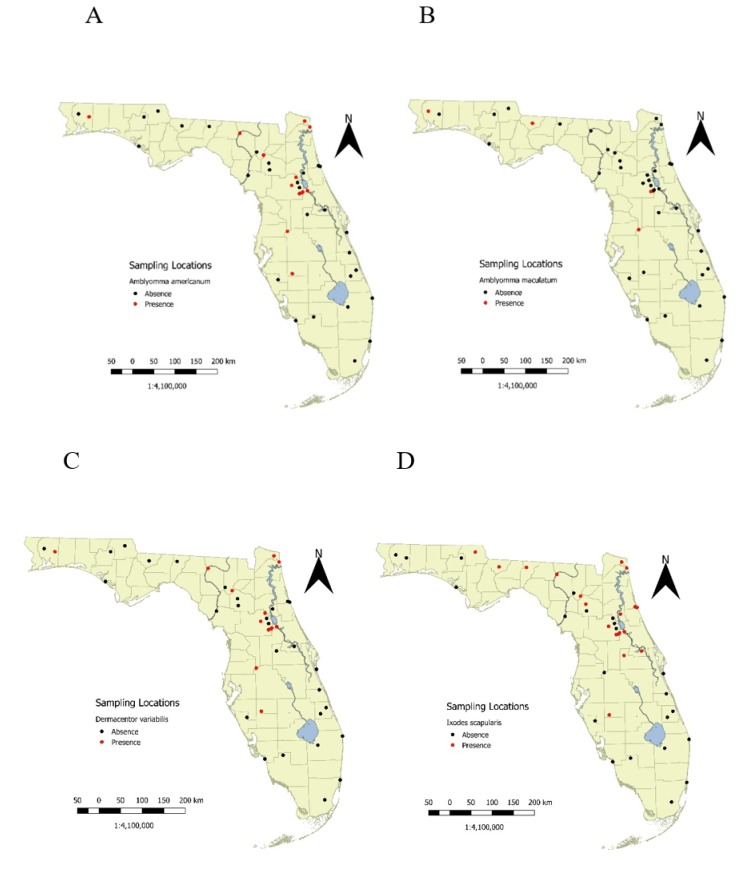
A–D. Sites with ticks present (red) or absent (black) during surveys between 2015–2018. (**A**) *A. americanum*. (**B**) *A. maculatum*. (**C**) *D. variabilis*. (**D**) *I. scapularis*.

Of the 560 transects, one or more species of adult ticks were sampled on 152 (27.14%) of them. If any ticks were to be found, they were usually caught soon after sampling began. For example, at least one of the four tick species was identified at 80.8% of sites that eventually yielded ticks within the first two visits and 92.3% of sites where ticks were found yielded adults within only four visits. Eleven (26.8%) sites never yielded ticks on any of their transects. The numbers of visits at these locations ranged between eight and 11 surveys. These sites were predominantly in Southern Florida. Pin maps for each species showed substantial north–south trends in the occurrence of various species (Figure 2A–D). All four species were broadly distributed in the northern tier of counties and through the central region of the state. The proportion of sites with any of these species decreased in the south as well as in the panhandle, except for the far western part of the state (Figure 2A–D).

Alternative survey methods (dry ice and wild boar) to flagging indicated, that in our hands, flagging was a feasible strategy for surveying large regions repeatedly. A total of 59 transects in *A. americanum* sites were tested with dry ice (Figure 2A). Of these, 32/59 transects yielded 87 adult *A. americanum* (1.48 ± 0.35/survey; mean ± standard error) by flagging, while dry ice sampling adjacent to the transects yielded 32 adult *A. americanum* (0.54 ± 0.23/survey). At 63% of transects, the two methods agreed (either present or absence), while at 29% of transects *A. americanum* was present by flagging but was not detected with dry ice. A smaller fraction of sites (8%) had *A. americanum* found by dry ice but not flagging.

The limited survey of harvested wild boar was conducted in two counties of Southern Florida during 2018 to determine if flagging was inefficient at collecting ticks in this region [23,27]. The preliminary results indicated that surveys of wild animals, despite their challenges, did identify the presence (if not abundance or risk) of some ticks [6]. Of the 16 boar examined, 16 individual adult ticks were obtained, including 14 *A. maculatum* and two *D. variabilis*. This represented the largest acquisition of Gulf Coast ticks obtained during the study. The dog ticks were from a region where we had not successfully flagged this species. The number of ticks per boar ranged from zero (nine boar) to six (one boar). Thus, ticks were not frequently found on boar nor were they abundant, but they were documented.

**Figure 3:**
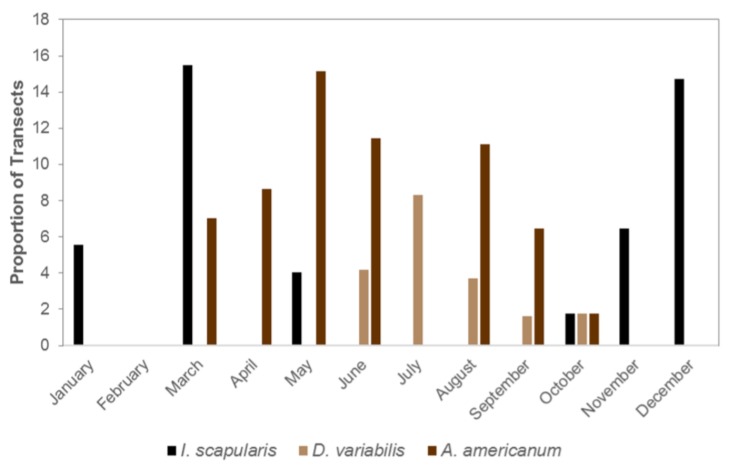
Proportions of transects collecting adult *I. scapularis* (blue), *D. variabilis* (orange) and *A. americanum* (gray), during each month, in Florida. No sampling was conducted in February.

The seasonal activity of questing adult *I. scapularis*, *D. variabilis* and *A. americanum* resulted in at least one of these species being active nearly the entire year (Figure 3). Too few *A. maculatum* were sampled to characterize their activity patterns. Adult *A. americanum* were collected between March and October, except for a brief period in the middle of the summer, while *D. variabilis* were first observed in May and last collected in October. Adult *I. scapularis* had a complementary questing period, first being collected in October and continuing through May the following year. For each of the species the monthly activity was statistically different from uniform, throughout the year (*p* < 0.001)

## 4. Discussion

The occurrence and geographic distributions of ixodid ticks in Florida has been studied, often through convenience sampling for more than a century [17,21,22,25,26]. As one of the states with the greatest extents of sub-tropical/tropical climates in continental North America, it represents a gateway transitional region into more temperate regions of the country. Additionally, during the past two decades, the region has undergone a tremendous human population influx which has influenced the potential impact of TBPs on resident and visiting populations and places visitors and workers at risk.

The U.S. CDC has undertaken efforts to elicit standardized protocols for surveying selected ixodid tick species [6]. Of special importance, it outlines how differences in methods and approaches impact the types of conclusions that can be drawn from the collected data. Thus, for example, collections from wild animals meet the objective of documenting occurrence within a region (e.g. state or county) and documenting the occurrence of TBPs, but they are not acceptable for estimating the prevalence of the TBPs (as one metric of risk), density of host-seeking (infected) nymphs (DON/DIN) or density of (infected) females (DOF/DIF), or the phenology of the tick species. In contrast, flagging/dragging meets all these goals [6]. The impact of survey methods (and estimated geographic extents) on collected tick species (Table 1) is evident in the state. Although only one of the studies compared survey methods at a single location [27,28], differences in species composition were striking depending on the survey method. It was most evident in surveys for *R. sanguineus* in the state. Among all the recent studies, only Burroughs and colleagues [25] found any individuals of this species—and they were reported in large numbers. This was the only study that used domestic animals as their survey method, and it was not surprising that nearly all *R. sanguineus* were removed from domestic dogs. Thus, no one method meets all the needs of survey sensitivity for all species. This makes cross-species comparisons of differences in TBD risks for different pathogens transmitted by different vectors that are sensitive to applied survey methods difficult. Thus, explicit descriptions of methods remain critical, and risks for different TBDs are likely constrained to descriptions of geographic variation for individual species.

Flagging was chosen for this study as most closely matching CDC recommendations for generating the greatest range of information to assess risk. In addition, it came closest to activities many outdoor workers and recreation participants might experience walking through various outdoor environments, as a surrogate of exposures [39]. It also provided, in our experience, the best tradeoff in survey speed and data aggregation, allowing the most efficient survey strategy. Comparison with other methods, performed by us, suggested that flagging for questing ticks was generally satisfactory, with some caveats. The time required to flag each 150 m transect meant flagging sampled more local habitat compared to the stationary dry ice trap. Thus, it is unsurprising that, on average, more *A. americanum* were flagged compared to dry ice. Conversely, surveys using wild animals, despite limitations noted by CDC, provided important species distribution records that flagging did not provide. Thus, even though the effort was limited, wild animal surveys provided the only records of *A. auricularium, A. maculatum* and *D. variabilis* in a region of the state where active flagging failed to yield specimens. The limitations of wild animal data concerning the uncertainty of spatial location/resolution, the prevalence of TBP’s and density estimates remained.

Another limitation of presenting the flagging results as pin maps (Figure 2A–D) is that even with the effort invested, the local data represent an exceptionally small geographic extent surveyed within the region. Using the species record locations, alone, conflate both the environmental and ecological factors, limiting species ranges with the accessibility of other areas that were not sampled due to time, access or other resource limitations. Thus, SDMs attempt to provide rational methods to estimate species geographic ranges by extrapolating local environmental conditions with, at least, presence data coupled to more extensive environmental conditions that might be associated with tick presence/abundance. As such, SDMs may provide a resolution of the apparent absence of ticks from flagging in Southern Florida [16]. Are the environmental conditions in this region similar enough to the north-central portion of the state that we would expect ticks to be present? Or, are the environmental metrics in the region so different that we predict the species should be absent even though wildlife surveys show that the ticks do occur?

Some of the earliest SDM efforts for ticks and TBD’s relied on statistical general linear models, such as logistic, multiple and Poisson regression [41,42]. During the past quarter of a century, these approaches have been supplemented (or supplanted) by other approaches by addressing the practical challenges that real-world data collections impose on methodological underpinnings. Probably the first of these, and the most challenging remains the absence of ‘absence’ data. Archived data, such as museum collections [4], or citizen science contributions [43], often lack information on locations that were ‘sampled’ but no ticks were collected. Thus, identifying species ecological limits is challenging with these data.

Many methods, such as Maximum Entropy [44], GARP [45], Machine Learning [46] approaches have used different assumptions to resolve various challenges imposed by parametric models [47]. Diagnostics incorporated into the model development are intended to evaluate the quality of the model outputs compared with the observations, and usually involve randomizng data subsets, re-estimating the model and comparing the similarities of the outputs. While these processes provide important information on the internal consistency of the model construction, they do not address sampling bias that makes extrapolation (external validity) a challenge [15]. This has been a concern that geographers have tried to explore repeatedly since SDMs became of increasing importance in a diverse array of fields [15]. The consensus of these studies is that internal validity studies do not rescue concerns about extrapolation to unsampled regions when bias occurs (the extent of bias needed to significantly impact predictions has not been defined).

Thus, sampling design, rather than post hoc adjustments, to reduce or at least better define the types of bias inherent in survey studies becomes important and this requires the expertise and experience of field biologists and acarologists [43]. In this study, strata associated with climate and landcover were presumed to be important to species distribution based on previous ecological studies [4,5,35]. Attempts to sample proportional to coverages was intended to better estimate the extent of exposure that human and domestic animal populations would experience within the state. The goal of the survey was to establish a format to deal with external validation issues of SDMs for tick vectors in Florida. Specifically, it forms the basis for the presumably ‘best’ validation approach for predicted vector distribution models [15,44]. In subsequent surveys, models from these data form the basis for forecasts that will either be validated (or not) by repeating the surveys both in some of the previously sampled sites, to assess temporal stability, as well as in previously unsampled locations to assess the generalizability of the SDMs predictions.

## 5. Conclusions

A survey strategy primarily targeting adult, questing ixodid ticks was performed in mainland Florida, USA, from late 2015 to late 2018. Flagging on paired transects in the major biotopes was performed. Sampling was stratified by the six climatic zones of the state and was performed proportional to the land cover classes. The goal was to better understand the geographic extent of major vector species throughout the state while reducing survey bias. Four species were identified; *A. americanum*, *A. maculatum*, *D. variabilis* and *I. scapularis*. There was broad geographic heterogeneity in questing tick activities with all four species being found primarily in the north-central and central portions of the state, with few numbers in the panhandle and southern reaches. Flagging was generally found to be a satisfactory survey method, outperforming dry ice trapping but being more insensitive than wild animal checks in the southern region. *A. americanum* and *I scapularis* were the most frequently sampled species and intensities of these species on transects were aggregated at very local scales, with distances to independence of approximately 250 m and 100 m, respectively. Adult *I. scapularis* had seasonal activity that complemented *A. americanum* and *D. variabilis* so that adult ticks were active nearly year-round. Sampling strategies for tick surveillance have inherent challenges that impact results associated with surveillance. Many analytical methods for species occurrence/abundance are blind to issues of data quality input to the models. Plans for surveys should avail themselves of the extensive experience that epidemiologists have generated on controlling, reducing or recognizing potential bias in their studies.

## Figures and Tables

**Table 1:** Sample sizes from current survey and other, recent studies in Florida using different collecting methods. Numbers of adult and nymphal ticks are shown. Nymphs were primarily reported as *A. americanum*.

Study Climate Zones [36]	Method	*A. americanum*	*A. maculatum*	*D. variabilis*	*I. scapularis*	*R sanguineus*	Immatures
Current 1–6	Flag	501	8	50	234	0	1240
Merrill [27] 4	Flag/Boar	0/0	15/979	0/12	13/8	0/0	2958/1
Hertz [26] 1–5	Wild animals	3114	317	125	592	0	Not done
Burroughs [25] 1–6	Domestics	51	4	10	29	1243	‘Rare’
Cilek [24] 1	Flag	102	27	1	653	0	‘Rare’
Greiner [23] 5	Boar	3	287	2423	52	0	0

## Supplementary Materials

The following are available online at https://www.mdpi.com/2075-4450/10/8/235/s1, Figure S1: Locations of survey sites (black dots) within mainland Florida.
