# Supplementary figures and images for: Standardized Ixodid Tick Survey in Mainland Florida
Source: Insects. 2019 Aug 1;10(8):235. doi: 10.3390/insects10080235 (PMC6722812; doi:10.3390/insects10080235)

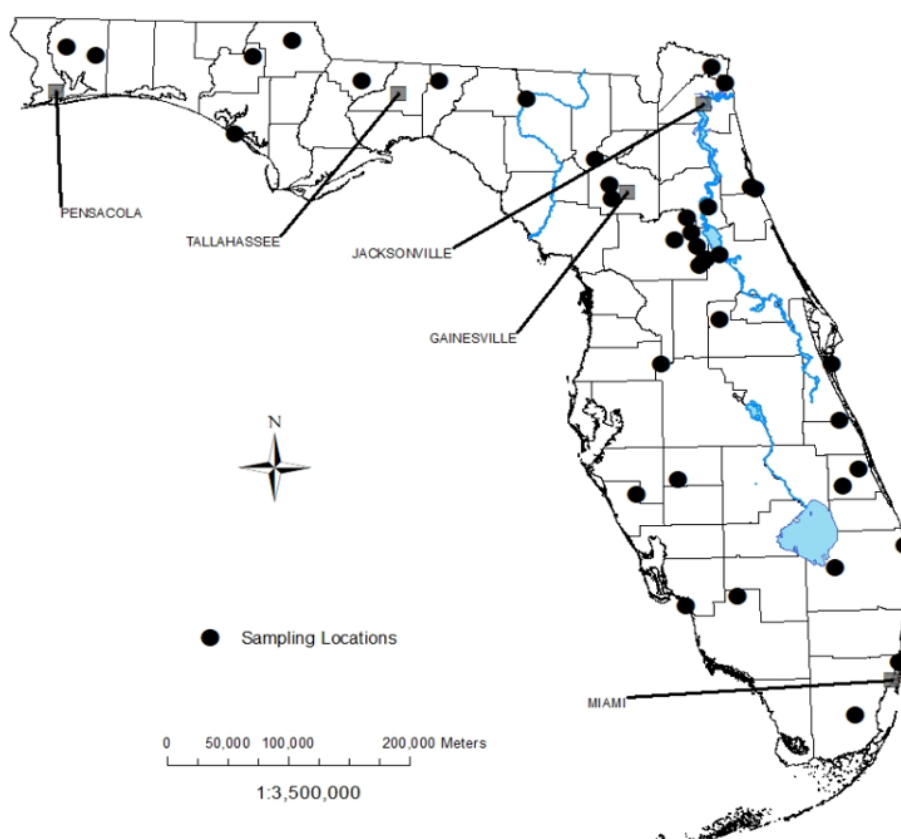

**Figure S1.** Locations of survey sites (black dots) within mainland Florida.

Supplement: Supplementary file 1 [file insects-10-00235-s001.pdf]
